# Changes in the Relative Inflammatory Responses in Sheep Cells Overexpressing of Toll-Like Receptor 4 When Stimulated with LPS

**DOI:** 10.1371/journal.pone.0047118

**Published:** 2012-10-04

**Authors:** Shoulong Deng, Qian Wu, Kun Yu, Yunhai Zhang, Yuchang Yao, Wenting Li, Zhuo Deng, Guoshi Liu, Wu Li, Zhengxing Lian

**Affiliations:** 1 Laboratory of Animal Genetics and Breeding, College of Animal Science and Technology, China Agricultural University, Beijing, P.R. China; 2 School of Biological Science and Medical Engineering, Beijing University of Aeronautics and Astronautics, Beijing, P.R. China; 3 College of Animal Science and Technology, Northeast Agricultural University, Harbin, P.R. China; 4 National Key Lab of Agrobiotechnology, Beijing, P.R. China; 5 College of Animal Science and Technology, Anhui Agricultural University, Hefei, P.R. China; 6 Department of Animal Science, Oklahoma State University, Stillwater, Oklahoma, United States of America; Auburn University, United States of America

## Abstract

**Background:**

Many groups of Gram-negative bacteria cause diseases harmful to sheep. TLR4 is an important Toll-like receptor (TLR) which responds to common Gram-negative bacterial infections. Activation of TLR4 leads to the induction of inflammatory responses, which is a linkage between the innate and adaptive immune systems. A vector pTLR4-3S was constructed to overexpress TLR4 gene in sheep. In this study, effects of TLR4 overexpression on inflammation response under LPS stimulated were addressed in vivo and in vitro.

**Methodology/Principal Findings:**

Sheep fetal fibroblasts were transfected with expression vector pTLR4-3S. Transgenic sheep were produced by microinjection of the constructed plasmids into fertilized eggs. Fetal fibroblasts, monocyte-macrophage and fibroblasts isolated from the transgenic sheep were stimulated by LPS. After that immunoactive factors (TNF-α, IL-10, IL-6, IL-8, IFN-γ), nitric oxide, phagocytize ability and adhesion were detected. Furthermore, transgenic sheep were intradermal injected of LPS in ear and observed pathological changes by HE strain. Overexpression of TLR4 gene was observed on transgenic cells and individuals. In vitro, TLR4 overexpression transgenic cells secreted Th1 and Th2 inducing cytokines with a strong LPS mediated inflammation response and promoting the secretion of nitric oxide, and then recovered to initial level. The phagocytosis index of monocyte/macrophage in transgenic sheep was higher than that of non-transgenic sheep (*P*<0.05). In vivo, tissue sections showed that transgenic individuals launched inflammation response more quickly.

**Conclusions/Significance:**

Overexpression of TLR4 in transgenic sheep enhanced the clearance of invaded microbe through secretion of cytokines, activation of macrophage, oxidation damage and infiltration of neutrophil.

## Introduction

About 90–95% of Gram-negative bacteria are considered to be harmful to their hosts. Many intracellular bacteria, such as, *Brucella*, *Tubercle bacilli*, and *Salmonella*, are pathogenic to both animals and humans and they can be transmitted from animals to humans. These intracellular bacteria are difficult to clear by host immune system and they can cause several syndromes, even death. This is due to inhibit macrophage activity and prevent the formation of phagolysosomes. Infected macrophages can sense the digested product of intracellular bacteria, present antigens, and secrete interleukin-12 (IL-12), which promotes differentiation from Th0 to Th1 is impaired. Interferon-gamma (IFN-γ) and CD40L are expressed in Th1 activated macrophages. These induce breath-bursts to kill the intracellular bacteria in the macrophages. Gram-negative bacteria share a similar endotoxin, lipopolysaccharides (LPS), which cause severe syndromes. Large amounts can even cause endotoxemia and septic shock during later stages of infection.

Toll-like receptors (TLRs) are a class of type I proteins, playing key roles in innate immune. They detect the invading pathogen and to launch pathogen-killing reaction [1]. TLRs are conserved from drosophila to humans. These receptors are very similar, structurally and functionally. They recognize the pathogen-associated molecular patterns (PAMPs) expressed on infectious microbes and mediate the production of cytokines necessary for the development of effective innate immunity [2]. TLR4, which recognizes LPS and initiates a series of intracellular responses, invokes a vigorous cytokine response among immune cells against Gram-negative bacteria [3], TLR4 activates the MyD88- and TRIF-dependent pathways through nuclear factor-κB (NF-κB)–associated signaling events [4]–[5]. It triggers tumor necrosis factor alpha (TNF-α) and it is associated with the activation of the pro-inflammatory cytokines that cause the inflammatory cascade reaction [6]. TLR4 also acts as a regulator in the immune system. The induction of antigen-presenting cell activation is mediated by acquired immune responses [7]. A mutation in the TLR4 gene was identified in a mouse hyporesponsive to LPS [8]. Bladder epithelial cells of extremely intractable urinary tracts were found to be strongly resistant to infection by most pathogens. These cells selectively exfoliated upon bacterial colonization and underwent re-epithelialization to reduce bacterial load in the bladder. After infection, bladder epithelia secreted large amounts of pro-inflammatory cytokines, such as IL-6 and chemokine, which are responsible for the vigorous neutrophil response and early clearance of infectious bacteria [9], [10]. IL-6 mobilizes and amplifies both local and systemic innate immune defenses against infection. It is up-regulated by the acute phase protein [11].

Overexpression of TLR4 in transgenic animals improved the disease resistance accompanying vigorous injury. In transgenic mice with different numbers of copies of TLR4 driven by its natural promoter, overexpression of TLR4 increased neutrophil recruitment, micro-vascular and alveolar epithelial repair caused by protein leakage, and the damage to the lung micro-architecture in a dose-dependent manner. This indicates that TLR4 has an important effect on acute response [12], to study the role of the TLR4, we cloned its cDNA. After LPS stimulation, the activity of monocytes/macrophages to phagocytize was detected. Changing levels of cytokine expression and the release of nitric oxide (NO) were monitored. In vivo, LPS was injected intradermal into the ears of sheep.

## Results

### TLR4 expression vectors validation in 293FT cell

**Figure 1:**
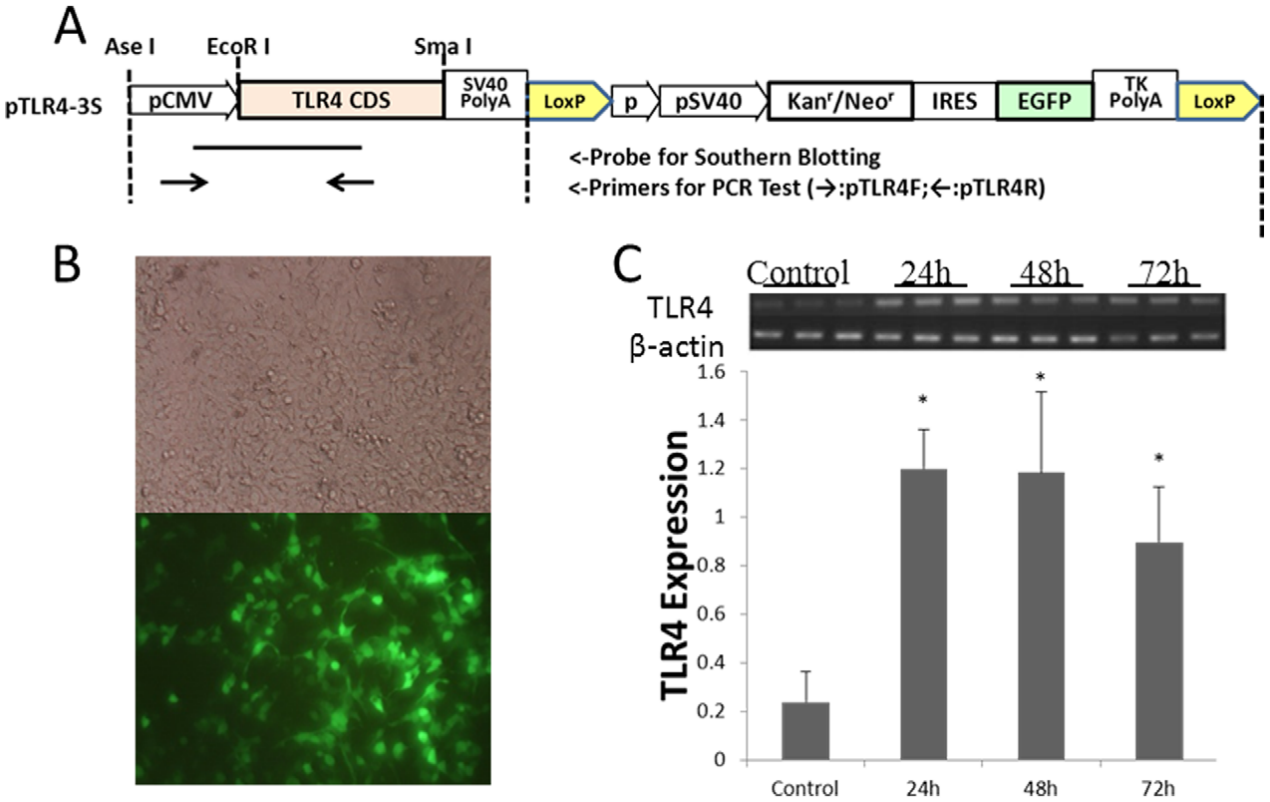
TLR4 expression vectors validation in 293FT cell. A) Construct pTLR4-3S vector; TLR4 expression structure in 293FT cell and its efficiency. B) Construct expressing green fluorescent protein in the 293FT cell (200×). C) pTLR4-3S transfected into 293FT cells. TLR4 expression was detected by RT-PCR. Gray value results confirmed TLR4 overexpressed for at least three days.

EcoRI and SmaI restriction enzymes were selected to ligate the whole coding sequence of sheep TLR4 with p3S-LoxP vectors. Vector pTLR4-3S was used for transient transfection to verify the efficiency of the vector by detecting fluorescent signal in 293FT (Fig. 1A and B). After transfection, real-time quantitative PCR was used. It showed that vectors could strongly drive TLR4 transcription, which peaked at 48 hours (Fig. 1C). This showed that these vectors could be used in functional studies of sheep TLR4 in vitro or in vivo.

### Effects of overexpression of TLR4 in fetal fibroblasts in vitro on the inflammatory reaction

**Figure 2:**
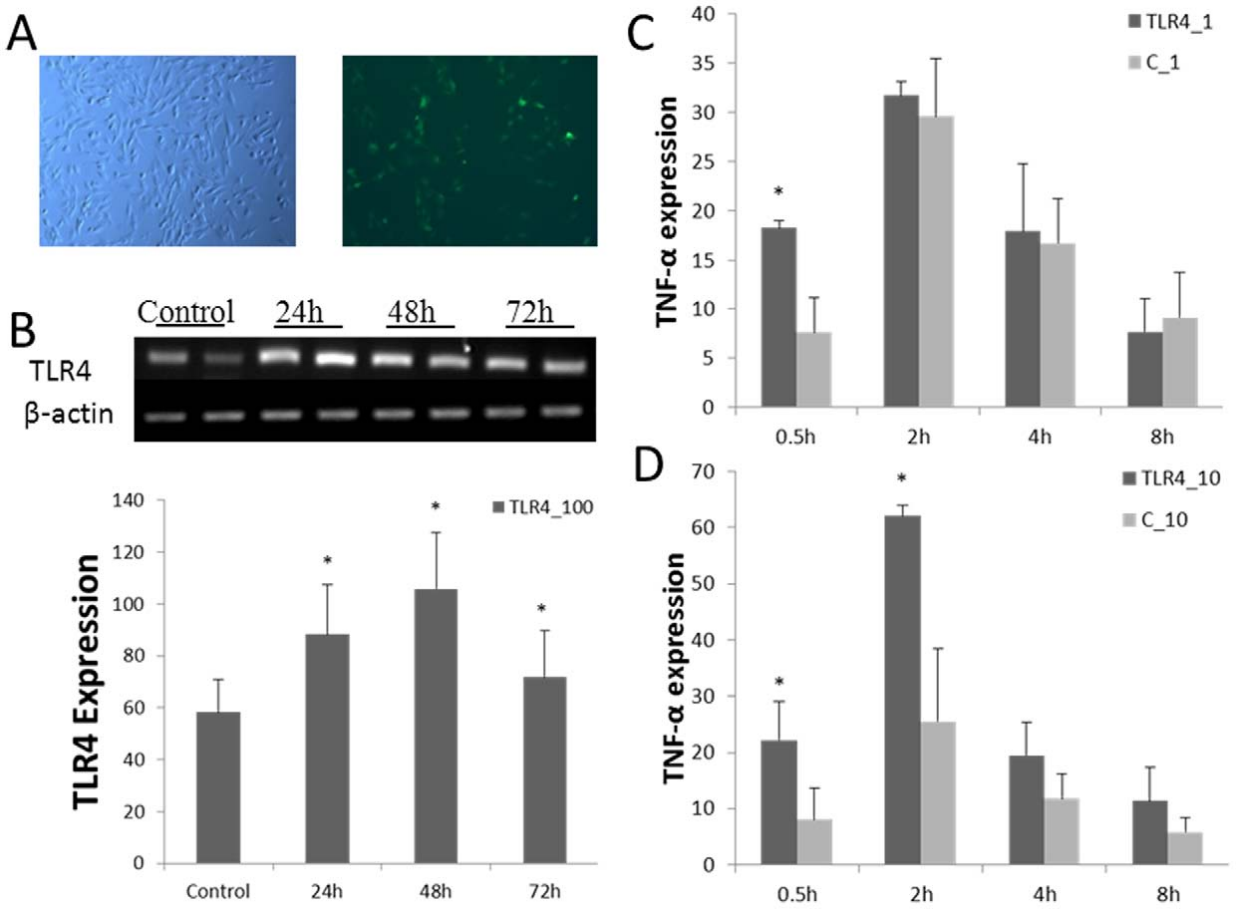
Effects of overexpression of TLR4 in fetal fibroblasts in vitro on the inflammatory reaction. A) Transient transfection of pTLR4-3S vector in the fetal fibroblast cell (100×). B) Show TLR4 overexpressed in fetal fibroblasts by transient transfection. C) and D) TNF-α transcriptional level under LPS (1 ng/mL, 10 ng/mL) stimulation. In the overexpression group, the subjects' immune systems quickly responded to stimulation. In graph: C =  subjects transfected with p3S-LoxP fetal fibroblasts, subjects transfected with TLR4 = pTLR4-3S fetal fibroblasts. Data shown are means ± SE. * Values within the same concentration of LPS with differ significantly across different groups (*P*<0.05).

At 24 hours after transfection with p3S-LoxP (control group) and pTLR4-Trans (TLR4 group), TLR4 transcription level was up-regulated (Fig. 2A and B). TNF-α is a downstream cytokine of the TLR4 signaling pathway, and it is activated directly by NF-κB. It is often representative of the level of activation of the immune system. In this study, large amounts of TNF-α were transcribed 0.5 hours after LPS stimulation. For overexpression group, cells immediately responded to stimulation, even LPS at a low concentration (1 ng/mL). Under 10 ng/mL LPS stimulation, TNF-α transcription significantly increased 2 hours after stimulation (Fig. 2C and D).

**Figure 3:**
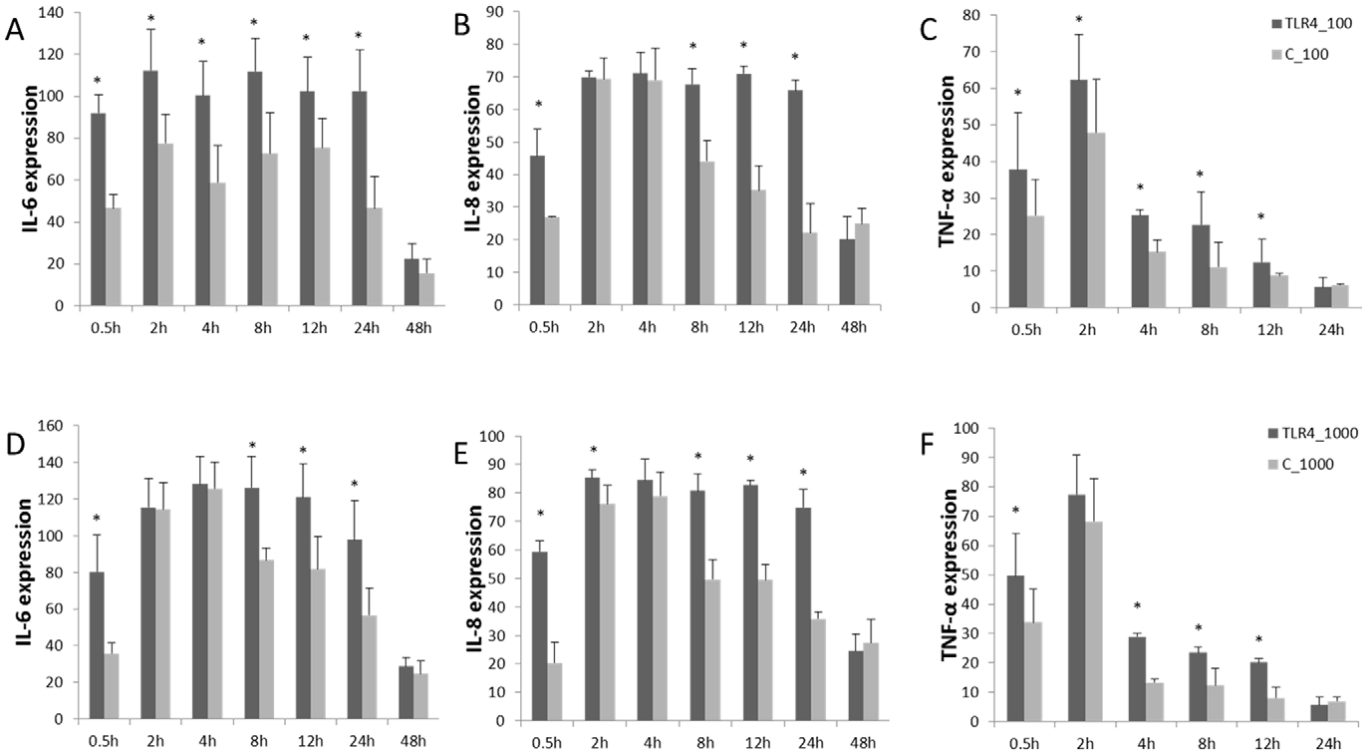
Pro-inflammatory cytokines expression of overexpression TLR4 in fetal sheep fibroblasts under LPS stimulation. A), B), and C) show the expression of IL-6, IL-8, and TNF-α under LPS (100 ng/mL) stimulation. D), E), and F) show expression of IL-6, IL-8, and TNF-α under LPS (1000 ng/mL) stimulation. In graph: C =  subjects transfected with p3S-LoxP transfected fetal fibroblasts, subjects transfected with TLR4 = pTLR4-3S fetal fibroblasts. Data are means ± SE. * Values within the same concentration of LPS differ significantly across different groups (*P*<0.05).

Sheep fetal fibroblasts were stimulated with 100 ng/mL and 1000 ng/mL LPS, and the expressions of cytokines were measured during different phases. Similar patterns were observed in both cases (Fig. 3). TNF-α increased significantly at 0.5 hours and reaching a peak at 2 hours. It declined dramatically till 4 hours and returned to normal levels at 24 hours. In addition, transcription levels of IL-6 and IL-8 were significantly up-regulated at 0.5 hours (*P*<0.05), reaching a peak at 4 hours, which was 2 hours later than TNF-α. Levels of IL-6 and IL-8 remained higher than in the control group, returning to average levels at 48 hours.

### Production of transgenic sheep overexpressing TLR4

**Figure 4:**
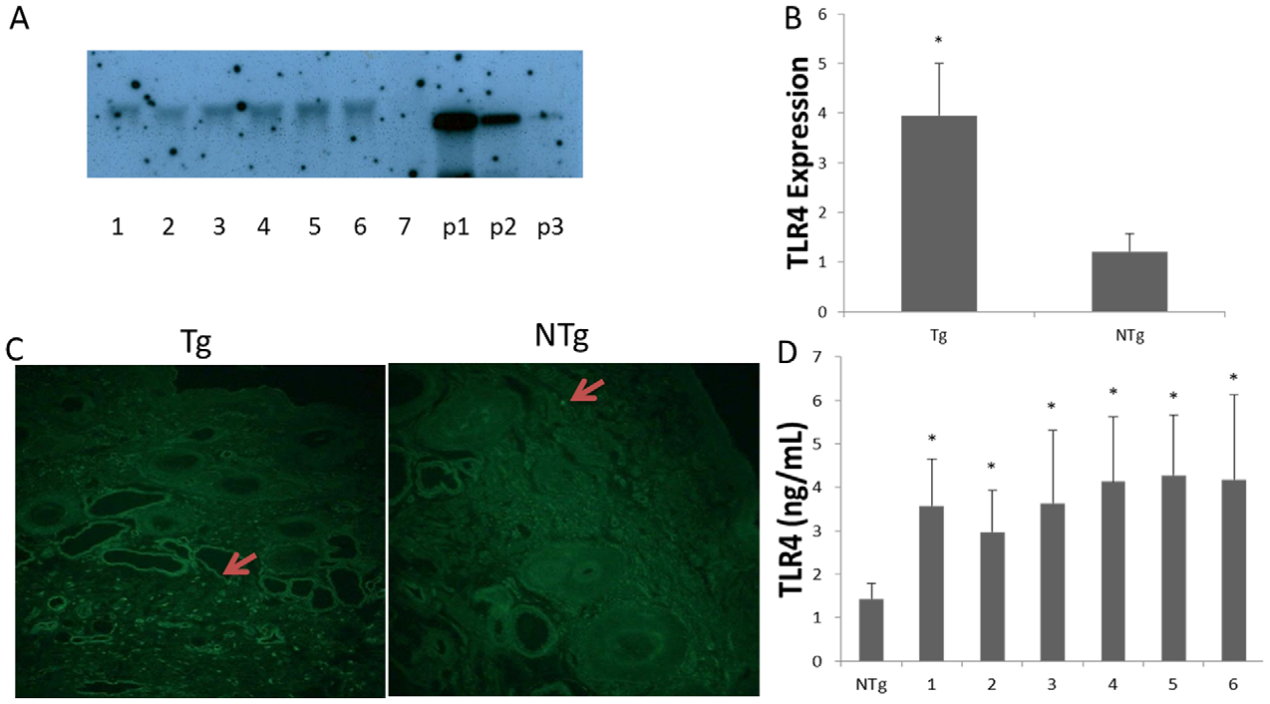
Production of TLR4 overexpression transgenic sheep by microinjection. A) Southern blot analysis. Samples from positive individuals (numbered 1, 2, 3, 4, 5, and 6) are shown with 7 as a negative control. p1, p2 and p3 are samples of transgenic vectors, here used as positive controls. B) Transcription of TLR4 in transgenic sheep. C) immunohistochemical staining showed more TLR4 expression in transgenic animals (100×). Sections were stained with TLR4-FITC (green). D) Protein levels of TLR4 in transgenic sheep. Tg  =  transgenic sheep, NTg  =  non-transgenic sheep. *Different letters indicate significantly different values (*P*<0.05).

Transgenic sheep were produced by microinjection. The ewes used in this experiment were 1 to 3 years old. In total, 51 sheep underwent superovulation, and 575 early-stage embryos were collected. Test microinjections were performed to optimize the efficiency concentrations of linearized DNA. A concentration of 5 ng/µL was found to have the most highly positive rate. After the linearized vectors were microinjected, 377 embryos were found to be transferable. There were 89 recipients. B-ultrasound diagnosis showed that 37 recipients were pregnant on days 30–35 after ET. The pregnancy rate of recipients was 41.57%. In total, 46 lambs were born. Southern blot analysis demonstrated that 13 lambs (7 female and 6 male) were found to be positive, carrying the exogenous TLR4. The Tlr4 Tg strains presented in their genomes various amounts of integrated Tlr4 copies: Four sheep had only 1 copy, five sheep had 2 copies, four sheep had 3 copies. The integration efficiency was found to be 28.26% (Fig. 4A and Table 1). In vivo, both real-time PCR (*P*<0.05) and immunocytochemical results revealed that TRL4 was overexpressed in transgenic individuals (Fig. 4B and C). TLR4 protein level of monocytes/macrophages was higher in the six transgenic male sheep than in the non-transgenic group by Elisa (*P*<0.05). No statistical difference between positive individuals (Fig. 4D).

**Table 1:** Production of transgenic sheep over-expressing TLR4.

Concentration of DNA	No. Donor	No. of micro-injection	No. of ET recipients	Pregnant rate (%)	Survival rate (%)	Positive rate (%) Southern
3ng/µL	39	202	50	46.00 (23/50)	86.21 (25/29)	28.00 (7/25)
5ng/µL	12	175	39	35.90 (14/39)	91.30 (21/23)	28.57 (6/21)
Total	51	377	89	41.57 (37/89)	88.46 (46/52)	28.26 (13/46)

Note: No.  =  number.

### Enhance phagocytosis and adhesion of monocytes/macrophages in sheep overexpressing TLR4

**Figure 5:**
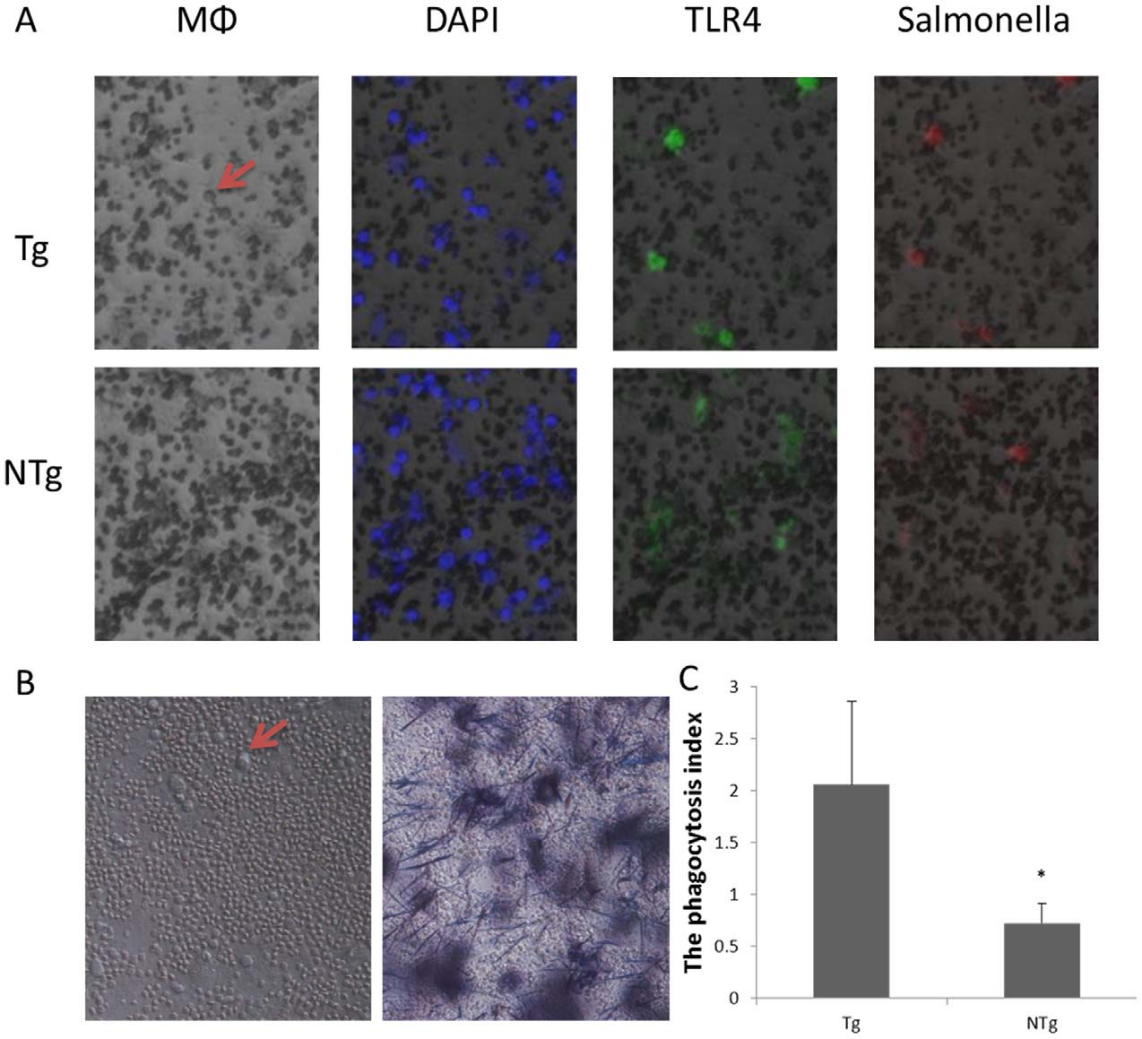
Phagocytosis and adhesion of monocyte/macrophage in Tg. A ) **monocytes/macrophages** (**arrows**) **are large cells** (**200×**)**, stained with DAPI** (**blue**)**, TLR4-FITC** (**green**)**, and Rhodamine B label** ***Salmonella*** (**red**)**.** B) The HCT8-MTT method was used to assess phagocytosis. C) The phagocytic index of transgenic group was higher than that of non-transgenic group. Tg  =  transgenic sheep, NTg  =  non-transgenic sheep. The results were means ± SE. *Different letters indicate significantly different values (*P*<0.05).

Immunohistochemistry was used to assess the capacity of *Salmonella* to adhere to target cells and to express TLR4 (Fig. 5A). The HCT8-MTT method was used to measure phagocytosis. In this experiment, sheep monocytes/macrophages were used. Tumor cells rich in mitochondria were strained by MTT incubated with monocytes and then the OD values of the dyed-tumor cells that being phagocytized by monocytes were measured. The transgenic group showed strong phagocytosis (*P*<0.05) (Fig. 5B and C).

### Ear fibroblasts and monocyte/macrophages from transgenic sheep evoked strong inflammatory response after with LPS stimulation in vitro

**Figure 6:**
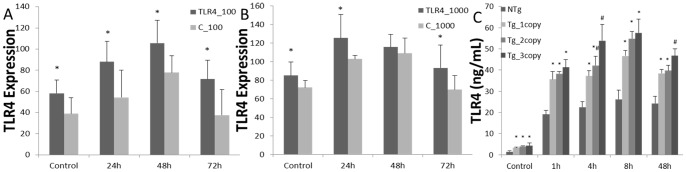
Expression pattern of TLR4 under LPS stimulation in monocytes/macrophages. Transcriptions pattern of TLR4 under 100 ng/mL and 1000 ng/mL LPS stimulation, respectively (A and B). Transgenic individuals were grouped corroding to exogenous TLR4 copy numbers. TLR4 protein levels were measured under 1000ng/mL LPS stimulation (C). Data were means ± SE. *^, #^ Values within the same time with different superscripts differ significantly between different groups (P<0.05). Same superscripts indicate no significantly different values between different groups (*P*>0.05). Tg  = Transgenic Sheep, NTg = Non-transgenic Sheep.

Absolute quantitative PCR was employed to study the TLR4 transcriptions Monocytes/macrophages from transgenic individuals were mixed and stimulated with 100 ng/mL and 1000ng/mL LPS, respectively. Tg group gave higher levels of TLR4 transcriptions under 100 ng/mL LPS stimulation (Fig. 6A). similar pattern was observed when cells challenging by 1000 ng/mL LPS(Fig. 6B). But the differences between Tg and NTg groups were relatively small. Transgenic male sheep were grouped according to the copy number: Tg_1 copy group (n = 1), Tg_2 copies group (n = 4), Tg_3 copies group (n = 1). Monocytes/macrophages from transgenic sheep were stimulated with LPS.

Monocytes/macrophages under 1000 ng/mL LPS stimulation, there was no significant difference in TLR4 protein expression of Tg groups at 0, 1 and 8 hours. Tg_3 copies group expressed higher TLR4 levels than Tg_1 copies at 4 h and higher than the other two Tg groups at 48 hours. TLR4 protein level of NTg was shown significant lower expression than Tg groups at each time (Fig. 6C).

**Figure 7:**
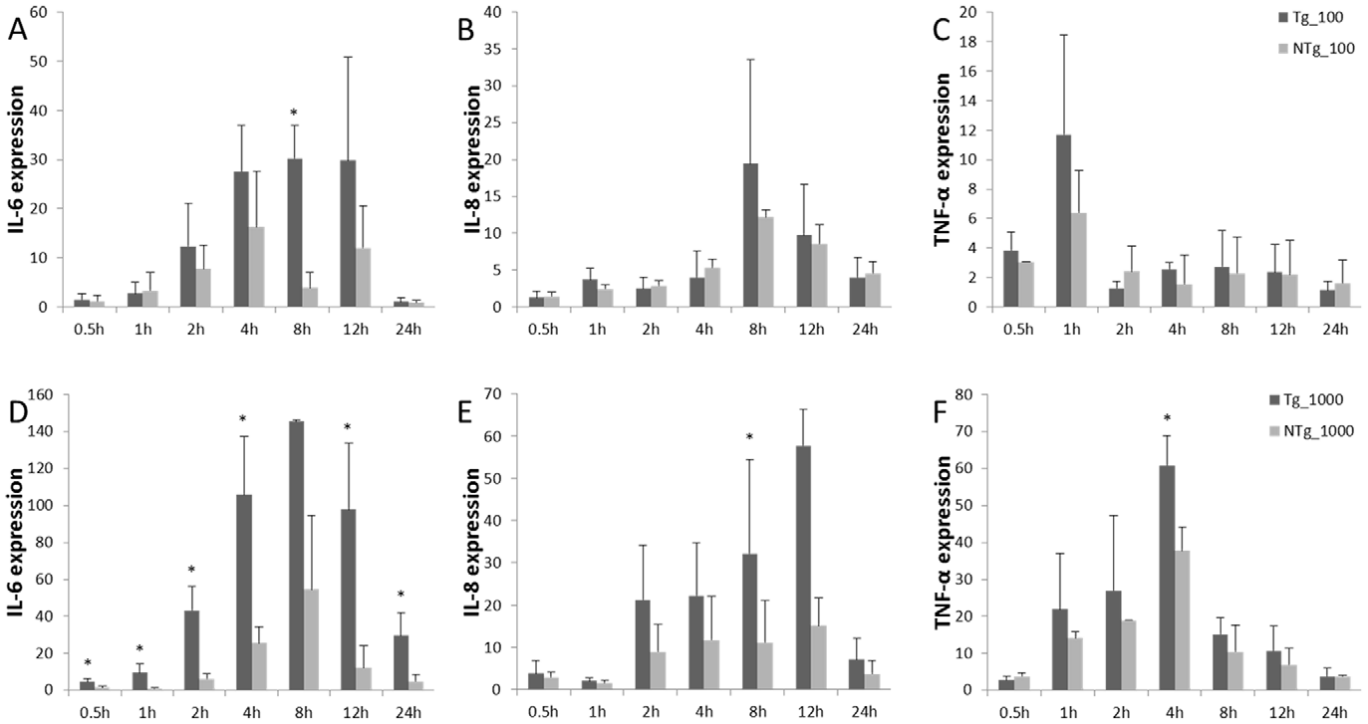
Expression pattern of fibroblast immune factor (IL-6, IL-8, TNF-α) under LPS stimulation and the expression pattern of IL-6, IL-8, and TNF-α under 100ng/mL LPS stimulation (A, B, and C). Expression patterns of IL-6, IL-8, and TNF-α under 1000 ng/mL LPS stimulation (D, E, and F). Data are means ± SE. * Different superscripts indicate significantly different values between different groups (*P*<0.05). Tg  =  transgenic sheep, NTg  =  non-transgenic sheep.

Fibroblasts were stimulated with LPS, and levels of TNF-α, IL-6, and IL-8 expression were assessed (Fig. 7). Under LPS stimulation, IL-6, IL-8, and TNF-α expression was more pronounced in the transgenic group than in the non-transgenic group, on average. For transgenic animals, expression of IL-8 and TNF-α in cell stimulated with 100 ng/mL LPS peaked faster than in cells stimulated with 1000ng/mL LPS. Rapid up-regulation of IL-6 expression was observed at 0.5 hours after stimulation with 1000 ng/mL LPS, and it lasted for 8 hours after stimulation. A similar pattern was observed with IL-8 expression. TNF-α expression was up-regulated to dramatically higher levels than non-transgenic animals by 4 hours after stimulation. This expression had rapidly declined by 8 hours after stimulation. Expression of all three cytokines declined to initial levels within 24 hours of stimulation. All of these findings indicate that transgenic animals can respond rapidly to bacterial infection and that they do so by releasing more cytokines than non-transgenic animals.

**Figure 8:**
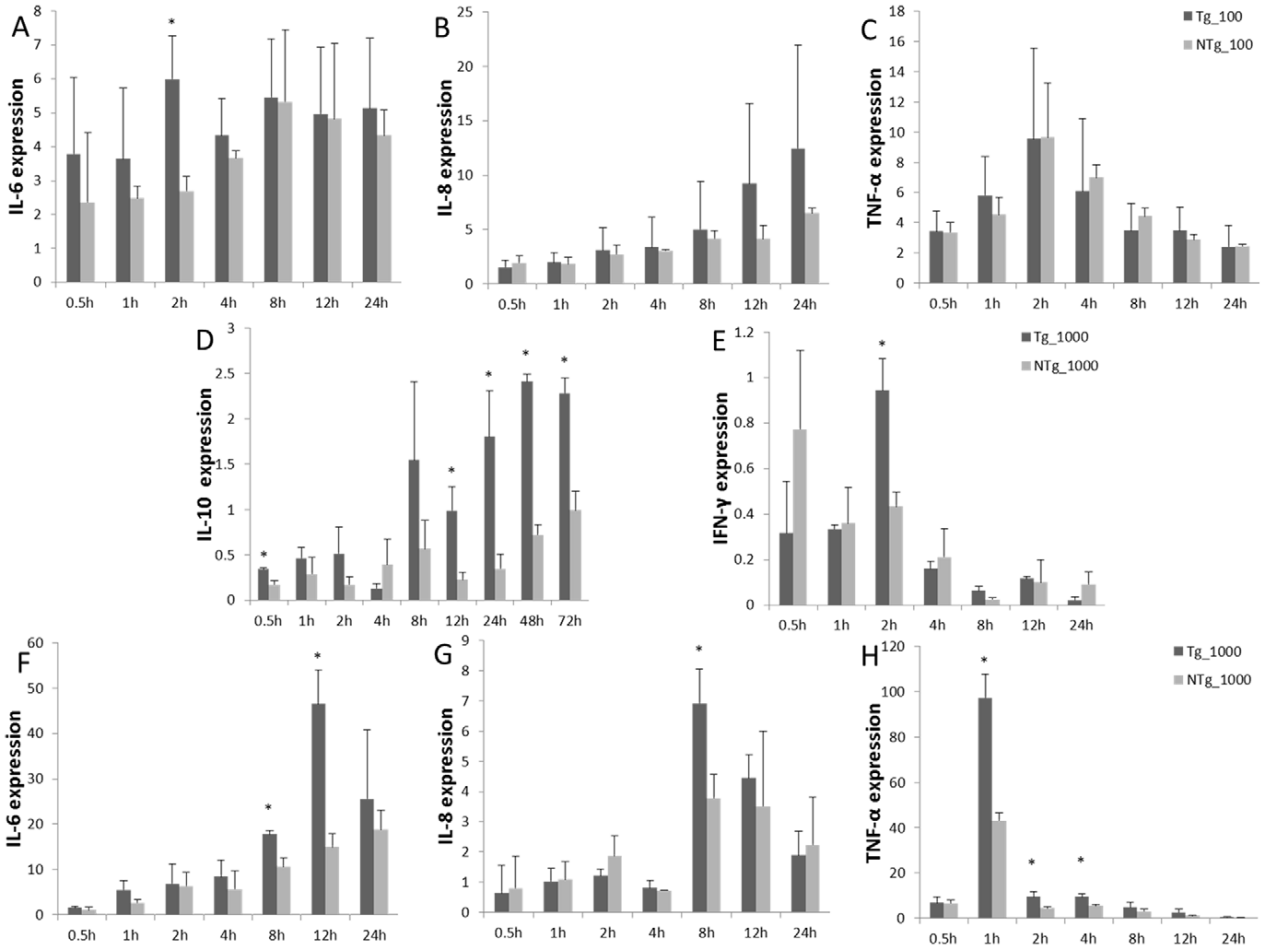
Expression pattern of monocytes/macrophages released immune factor (IL-10, IFN-γ, IL-6, IL-8, TNF-α) under LPS stimulation. Expression patterns of IL-6, IL-8, and TNF-α under 100 ng/mL LPS stimulation (A, B, and C). Expression patterns of IL-10, IFN-γ, IL-6, IL-8, and TNF-α by Tg and NTg under 1000 ng/mL LPS stimulation (D, E, F, G, and H). Tg  =  transgenic sheep, NTg  =  non-transgenic sheep. Data are means ± SE. *Different superscripts indicate significantly different values between different groups during the same time period (*P*<0.05).

Monocytes/macrophages were challenged with LPS, and levels of TNF-α, IL-10, IL-6, IL-8, and IFN-γ transcription were measured (Fig. 8). In the transgenic group, under 100 ng/mL LPS stimulation, IL-6 expression remained relatively high throughout the study. This differed significantly from the non-transgenic group at 2 hours post-stimulation. At no point in the study did the transgenic and non-transgenic groups differ with respect to expression of IL-8 and TNF-α. Expression of IL-6 and IL-8 remained high, indicating that the inflammation reaction was ongoing. Under 1000 ng/mL LPS stimulation, IL-6 expression was up-regulated, and it peaked 12 hours after stimulation, followed by a decline. The expression of IL-8 continued to increase in both transgenic and non-transgenic animals. The highest levels of TNF-α and IFN-γ expression were observed 2 hours and 1 hour after stimulation, respectively, after which the expression of both declined immediately. The transient expression of TNF-α and IFN-γ helped to prevent over-inflammatory reaction. IL-10 expression was shown to increase significantly by 0.5 hours after stimulation in transgenic cells and tended to be up-regulated during the experiment. This indicates that anti-inflammatory factors were released.

### Overexpression of TLR4 induced strong oxidative injury by secret NO of monocyte/macrophage in transgenic sheep

**Figure 9:**
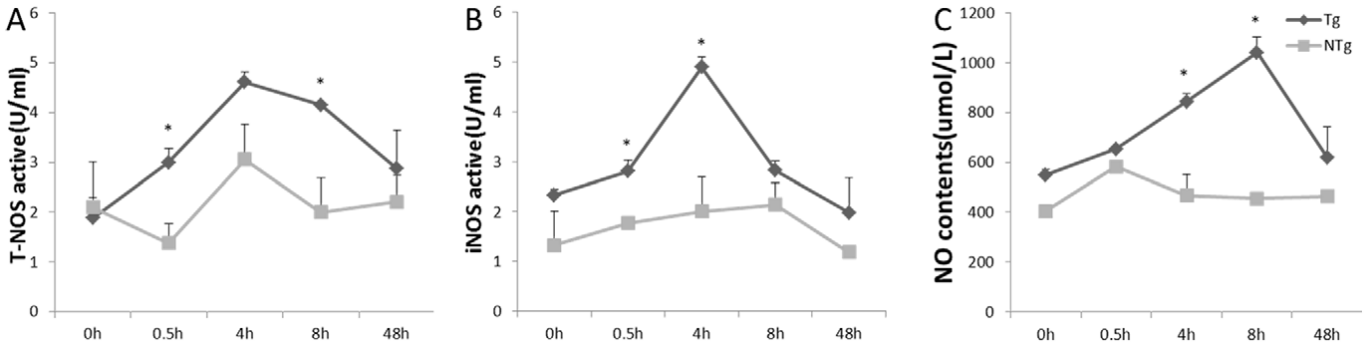
The activities of T-NOS, iNOS, and the contents of NO expression of monocytes and macrophages under LPS stimulation at 1000ng/mL. Tg  =  transgenic sheep, NTg  =  non-transgenic sheep. Data are means ± SE. *Different superscripts indicate significantly different values between different groups during the same time period (*P*<0.05).

NO plays an important role in killing the invaded microbes in a non-specific manner. Levels of NO, T-NOS, and active iNOS are shown in Figure 9. For transgenic sheep, similar patterns were observed – a dramatic rise and a rapid decline. Expression of iNOS was up-regulated at 0.5 hours post-stimulation and a 2-fold increase was observed over the non-transgenic group by 4 hours post-stimulation (*P*<0.05). The pattern of NO secretion was similar to that of IL-6 and IL-8.

### Rapid neutrophil infiltration in the ear of transgenic sheep after stimulation with LPS

**Figure 10:**
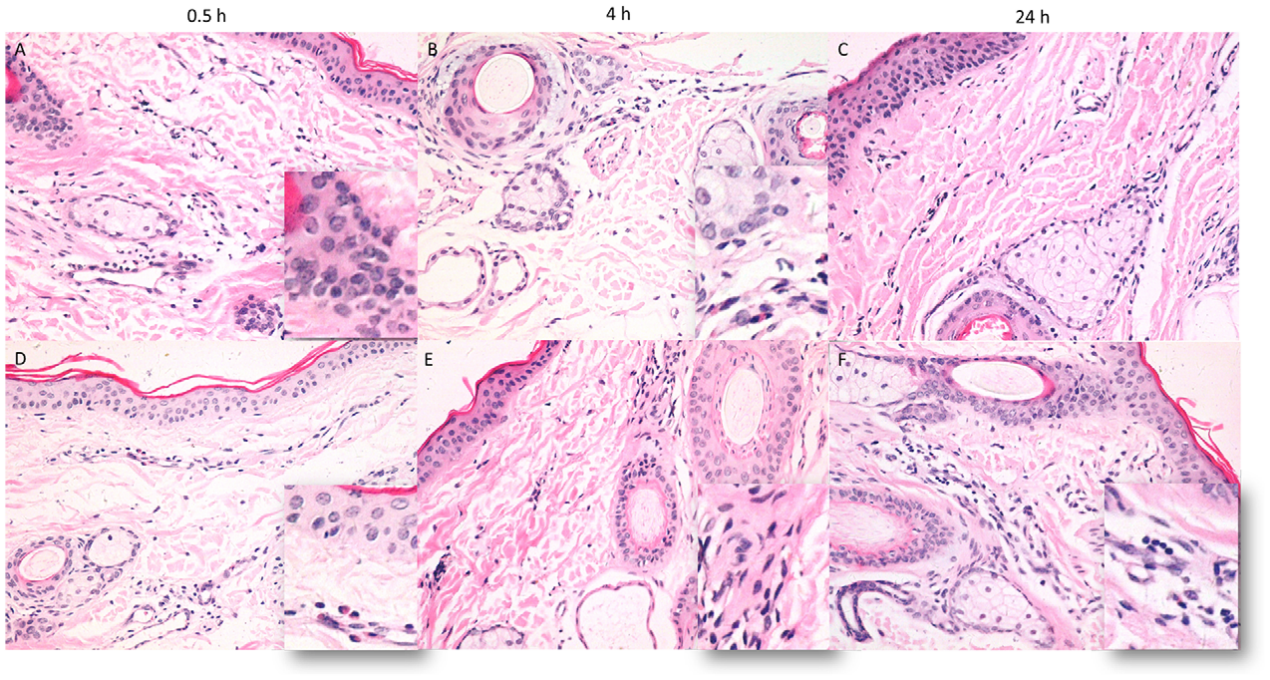
Pathologic observation of LPS in sheep. Histological study of ear tissues (HE staining, 200×). Tg  =  transgenic sheep (A, B and C), NTg  =  non- transgenic sheep (D, E and F).

Sheep ear tissue was exposed to 3 mg/mL LPS by intradermic injection. To assess HE staining, ear tissue sections were collected at different times from both transgenic and non-transgenic lambs (Fig. 10). In the transgenic group, inflammatory cell infiltration was observed around blood vessel dermis by 0.5 hours post injection. The horny layer was sloughed off and cell infiltration was observed by 4 hours post injection. By 24 hours post injection, no abnormalities were observed. In the non-transgenic group, only few eosinophil infiltrations were observed by 0.5 hours post injection. Cell components of the dermis were found to have increased by both 4 hours post-injection, and this effect lasted through 24 hours post-injection. Faster inflammatory reactions were observed in transgenic animals than in control animals.

## Discussion

Inflammation is a protective reaction that allows organisms to remove injurious stimuli and to initiate the healing process. Pathogen invasion itself can trigger innate immunity [13]. TLR4 has been identified as a main receptor for LPS. It has been found to be expressed in many types of cells. The TLR4 signaling pathway is involved in cytokine release, monocyte chemotaxis and cell infiltration that produce disease-resistant effects [14], [15]. Recently, studies have shown that overexpression of TLR4 can increase resistance to disease in mice [16]. TLR4 mutant mice appeared to have little or no response to LPS stimulation [17]. In this study, TLR4 cDNA was cloned, and a universal promoter was selected to produce transgenic sheep. Southern blot analysis confirmed that 28.26% of the offspring were positive. Results showed TLR4 was highly expressed in these transgenic sheep.

LPS is a natural immune activator. It can activate the mononuclear-phagocytic system, causing the release of a large number of cytokines and inflammatory mediators through TLR4 pathway [18]. It has been demonstrated that endotoxemia, colitis, and other inflammatory responses are associated with LPS-induced TLR4 expression [19]–[21]. Results indicated that TLR4 overexpression sheep quickly responded to LPS stimulation and increase the release of inflammatory cytokines, expanding the period of inflammatory response. TNF-α, a cytokine involved in TLR4 pathway, is one of the primary agents of the inflammatory response. It is important for pathogen resistance and in balancing the internal environment.

In this study, TLR4 overexpression enhanced the level of expression of IL-8, IL-6, and TNF-α. IL-6 acts by promoting the differentiation and infiltration of activated macrophages and by up-regulating the expression cell-adhesion molecules, during immune response [22]. Under LPS stimulation, TNF-α and IL-6 increasing of expression levels were necessary for immune response [23]. As shown in one previous study, IL-6 was down-regulated in TLR4 knock-out mice [24]. The primary function of IL-8 was induction of chemotaxis in certain cells. It also activates neutrophils. Fibroblasts overexpressing TLR4 were able to response to low doses of LPS (1ng/mL, 10ng/mL). Meanwhile, in transgenic group, the transcription levels of inflammatory cytokines were higher than in the non-transgenic group. That would help the organism eliminate pathogens.

The large amounts of TNF-α can cause tissue damage by inducing a cascade of endogenous mediators [25]. The release of inflammatory cytokines needs to be strictly regulated to prevent over-inflammation. To avid over-reaction caused serious tissue damage, there are internal mechanisms playing either negative role in TLR4 pathway. In this study, the TNF-α transcription level returned to average by 24 hours post stimulation. This indicated that the immune response was under the control of an internal mechanism. Under 1000 ng/mL LPS stimulation, TNF-α transcription peaked by 1 hour post stimulation in transgenic monocytes. This is earlier than in fibroblasts. Different transcription patterns probably due to the different cells [**26]**, [27].

During the immune response, IL-6 and TNF-α are distinctive feature factors whose expressions are up-regulated [28]. Along with LPS stimulation, IL-6 and IL-8 expression were dramatically enhanced [29], [30]. In this study, IL-6, IL-8, and TNF-α expression were all found to be up-regulated at first, but they later dropped back to initial levels. This is similar to the results of a previous study on macrophages [31]. The release of inflammatory cytokines only lasts for a short while to protect tissues from overreaction. IFN-γ, an inflammatory cytokine secreted by Th1 cells, inhibits Th2 differentiation. Th2-related cytokines include IL-10. IL-10 is an inhibitor of immunity cell differentiation and immune response. In the transgenic group, IFN-γ increased under LPS stimulation. Soon after that, IL-10 transcription became up-regulated and this up-regulation lasted for at least 72 hours. IL-10 acts on antigen-presenting cells to inhibit the release of cytokines and regulates TH1/TH2 balance. Under LPS stimulation, both inflammatory and anti-inflammatory cytokines were expressed. This protected tissue from excessive inflammation.

Monocytes play an important role in non-specific and specific immunological responses, which protect organisms from pathogens. Phagocytosis is an important part of the innate immune response. Macrophages and monocytes take a portion of the debris left over from the digestion of a pathogen and present it as an antigen to the adaptive immune system [32]. Phagocytosis of apoptotic inflammatory cells is one of mechanisms by which inflammation is eliminated [33]. TLR4 signaling was found to be necessary and sufficient for phagocytosis by monocytes. Phagocytosis was found to be correlated to the immune reaction. The present study indicated that TLR4 overexpression increased the phagocytic capacity of monocytes and macrophages. LPS is recognized by TLR4, which causes the production of NO and the release of inflammatory cytokines, which in turn promote inflammatory cell infiltration. TLR4 up-regulates iNOS transcription [34]. In macrophages, iNOS production is a result of activation by endotoxins and cytokines. The generation of NO help host to kill and inhibit the growth of invading microorganisms and neoplastic tissue [35]. NO directly or indirectly killed or reduced replication of infectious agents, such as viruses, bacteria, protozoa, fungi, helminthes, TNF-α can promote the synthesis of NO [36], [37]. In this transgenic group, NO expressed more NO and peaked 8 hours after LPS challenge. At the same time as NO production, IL-6 and IL-8 transcription increased. This indicated that corresponding to IL-6 and IL-8, NO contributed to inflammatory and anti-inflammatory effects.

In summary, Under LPS stimulation, Overexpression TLR4 animals rapidly activated the TLR4 signaling pathway. And this might help host launched the immune response against pathogen invasion and infection.

## Materials and Methods

### Ethics statement

Superovulation, artificial insemination, intradermic injection, and blood collection were performed at the experimental station of the China Agricultural University, and the whole procedure was carried out in strict accordance with the protocol approved by the Animal Welfare Committee of China Agricultural University (Permit Number: XK662). Sheep spleens were obtained from the Hai Dian Yong Feng slaughterhouse, a local slaughterhouse in Beijing, P.R. China.

### Expression vector for TLR4

RNA was extracted from sheep spleens using an OMEGA kit. The TLR4 cDNA sequence was amplified using the TLR4 mRNA sequence (Genbank Accession No. AM981302). The TLR4 cDNA sequence was amplified using reverse transcript-PCR. For further experimentation, restriction sites of EcoRI and SmaI (NEB, Beverly, MA, USA) were added to primers. The primers were as follows: forward: ccg gaa ttc ATG GCG CGT GCC CGC CG; reverse: tcc ccc ggg gGG TGG AGG TGG TCG CTT CTT GC. The size of the amplified fragment was 2523bp. After double enzymes (EcoRI and SmaI) digestion, PCR products were connected to the vector p3S-LoxP to generate TLR4 expression vector pTLR4-3S. Then 293FT cells (Life Technologies) were transiently transfected with the pTLR4-3S. Cells were collected 24, 48, and 72 hours after transfection. The expression of TLR4 was analyzed using real-time PCR with TLR4 special primers. β-actin was used as an internal standard: (TLR4 F: CTG AAT CTC TAC AAA ATC CC, R: CTT AAT TTC GCA TCT GGA TA; β-actin forward: AGA TGT GGA TCA GCA AGC AG, reverse: CCA ATC TCA TCT CGT TTT CTG), Real-time PCR reactions were carried out with a Real Master Mix SYBR Green Kit (Tiangen, China) using MX300P (Stratagene) following protocol [38].

### Overexpression of TLR4 sheep fetal fibroblast cells stimulated with LPS

Fibroblast cells were isolated and cultured from 3 month spontaneously aborted sheep fetuses, DMEM/F12 (Gibco, Grand Island, NY, USA) medium containing 10% FBS (Gibco, Grand Island, NY, USA) were used. pTLR4-3S were transfected into sheep fetal fibroblasts using liposomes (Lipofectamin 2000, Invitrogen, Carlsbad, CA). Cells were treated with different concentrations of LPS (Sigma, Chemical Co., St. Louis, MO) (1 ng/mL, 10 ng/mL, 100 ng/mL, 1000 ng/mL), and collected at different times. TLR4, IL-6, IL-8, and TNF-α transcriptions were monitored by real-time PCR. Primers specific to TNF-α, IL-6, and IL-8 were used (TNF-α F: AAC AGG CCT CTG GTT CAG ACA, R: CCA TGA GGG CAT TGG CAT AC; IL-6 F: GAC ACC ACC CCA AGC AGA CTA, R: TGC CAG TGT CTC CTT GCT GTT; IL-8 F: TCC TGC TCT CTG CAG CTC TGT, R: GGG TGG AAA GGT GTG GAA TG).

### Production of transgenic TLR4 sheep

Superovulation and artificial insemination were performed in sheep. The estrous periods were synchronized with controlled internal drug-releasing insert (CIDR; Pharmacia and Upjohn Company, Rydalmere, NSW, Australia). For superovulation, sheep were treated with CIDR + FSH. Fertilized eggs were collected and microinjected linearized pTLR4-3S vector in vitro. 5pL vector solution was microinjected into embryo with 3 ng/µL and 5 ng/µL. Well-fertilized embryos were transplanted into the recipient oviducts within 1 hour, and each recipient was transplanted with 2–5 embryos. To detect transgenic offspring, genomic DNA was extracted from ears, and exogenous genes were analyzed by Southern blotting. We used the PCR method to make specific digoxigenin-labeled probes (Roche Diagnostics, Mannheim, Germany). The TLR4 probes used for southern hybridization were as follows: F: TAC GGT AAA CTG CCC ACT TG, R: ACC TGG AGA AGT TAT GGC TG. In brief, genomic DNA (20 ng/µL) was digested with VspI and SmaI (NEB, Beverly, MA, USA), fragments the size of 3187bp. ear tissue were made into paraffin sections and immunohistochemically stained for observation of TLR4 protein expression. TLR4-FITC antibody was also used (Abcam, Cambridge, UK).

Peripheral blood monocyte/macrophages from transgenic male lambs were collected and their TLR4 protein levels were examined by Elisa.

Transgenic male sheep were grouped according to the copy number. Peripheral blood monocyte/macrophage cells treated with LPS (1000 ng/mL) stimulation were collected at different times and examined TLR4 expression by Elisa (Shanghai, Xinle, China).

### Detection of the immune response in transgenic sheep

Peripheral blood monocyte/macrophages from 3-month-old transgenic male lambs were collected and their phagocytic ability was assessed. Sheep lymphocyte separation medium (TBD, Tianjin, China) was used to isolate the cells. Monocytes cultured in RPMI-1640 (Gibco, Grand Island, NY, USA) medium supplemented with 10% FBS. Medium was changed every 24 hours.

To assess phagocytosis, cells were cultured for 72 hours. Briefly, cells were incubated in 100 μL culture medium containing 0.5% MTT (Amresco, Solon, OH, USA) for 2 hours in 96 well culture plates. 20 μL MTT treated HCT-8 solution was added. After 10 hours of co-culture, all media were removed. Cells were washed with 150 mL DMSO to fully dissolve crystals. Enzyme-linked immunosorbent assay (Bio-Rad) was carried out. OD values were measured at 570 nm wavelength. The phagocytic index calculated as OD (MTT-HCT8)/OD (MTT).

Rhodamine B labeled *Salmonella* was added to the culture medium and incubated for 2 hours. Immunohistochemistry was used to investigate the ability of *Salmonella* cells to adhere to and express TLR4 protein in the monocytes/macrophage.

Fibroblasts and mononuclear cells from positive individuals were stimulated by LPS under different concentrations (100 ng/mL, 1000 ng/mL). Cells were collected at different times for RNA extraction. The transcription levels of many different cytokines were detected using real-time PCR. Primers of TLR4, IL-6, IL-8, TNF-α, IFN-γ, and IL-10 were as follows: IFN-γ F: ATA ACC AGG TCA TTC AAA GG, R: ATT CTG ACT TCT CTT CCG CT; IL-10 F: TGC TGG ATG ACT TTA AGG G, R: AGG GCA GAA AAC GAT GAC A. TLR4, IL-6, IL-8, and TNF-α primers are indicated above.

Monocyte/macrophage cells treated with LPS (1000 ng/mL) stimulation were collected at different times. The NO contents were examined by spectrophotometry in accordance with the manual supplied with the NO detection kit (Nanjing Jiancheng, China).

In vivo, positive individuals were intradermally injected with LPS (3 mg/mL) 100 μL into ear, tissue samples were obtained at 0.5, 4, and 24 hours post challenge. Paraffin sections were prepared for observation of inflammatory response using HE staining.

### Statistical analyses

All data were subjected to analysis of variance using the GLM procedures of the statistical analysis system (SAS Institute, U.S.). All data are expressed as mean ± SEM. Differences were considered significant at *P<0.05*.
